# Supplementary material for: New insights into the design of conjugated polymers for intramolecular singlet fission
Source: Nat Commun. 2018 Jul 31;9:2999. doi: 10.1038/s41467-018-05389-w (PMC6068183; doi:10.1038/s41467-018-05389-w)
Supplement: Supplementary file 1 — Supplementary Information [file 41467_2018_5389_MOESM1_ESM.pdf]

Supplementary Information

New insights into the design of conjugated polymers for intramolecular singlet fission

Jiahua Hu,[1] Ke Xu,[1] Lei Shen,[1] Qin Wu,[2] Guiying He,[1] Jie-Yu Wang,[3] Jian Pei,[3] Jianlong Xia,[1]* Matthew Y. Sfeir[1,2*]

Bond Length Calculation of IIDDT-Me in the Ground and Triplet States

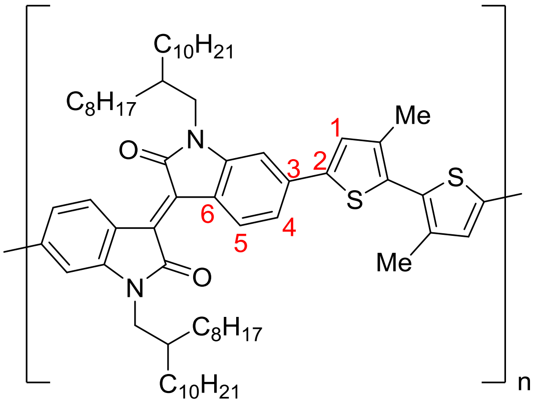

Supplementary Figure 1. IIDDT-Me with labeled atoms.

Supplementary Table 1. Selected bond lengths (in angstrom) in the optimized ground state (GS) and optimized triplet state (T_1) for IIDDT-Me. Carbon atoms are numbered as in Supplementary Fig. 1.

	IIDDT-Me		
	GS	T_1	Δ
C1-C2	1.375	1.384	-0.009
C2-C3	1.462	1.448	0.014
C3-C4	1.406	1.427	-0.021
C4-C5	1.391	1.375	0.016
C5-C6	1.401	1.418	-0.017

Second Derivative Analysis of the IIDDT-Me Absorption Spectrum

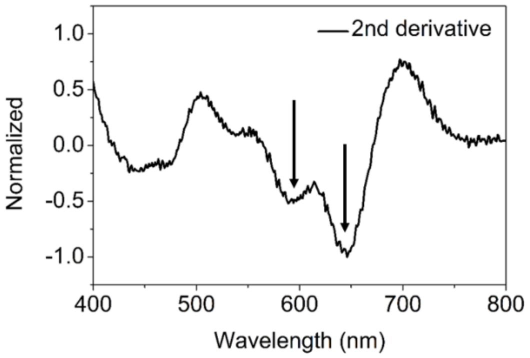

Supplementary Figure 2. The second derivative of UV-Vis absorption spectrum of IIDDT-Me in DCB solution. We have identified peaks at 645 nm (assigned to S_1) and 595 nm.

Supplementary Note 1. Comparison of the Free Triplet and Triplet Pair Decay

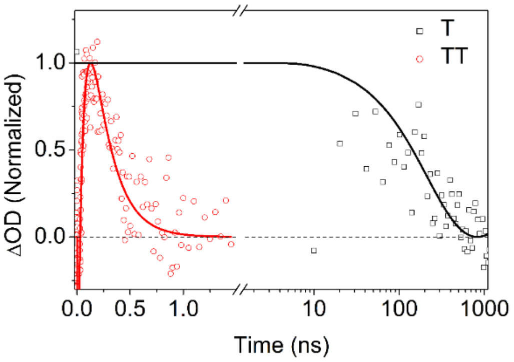

Supplementary Figure 3. Comparison of the lifetimes of the free triplet (black) and triplet pair state (red).

The lifetime of free triplet state is measured using triplet sensitization experiments following the scheme:

$$S_0(\text{anthracene}) \xleftarrow{k_{\text{aIC}}} T_\text{a}(\text{anthracene}) \xrightarrow{k_S} T_\text{p}(\text{polymer}) \xrightarrow{k_{\text{pIC}}} S_0(\text{polymer})$$

The time-dependent population of the triplet states of anthracene (T_a) and IIDDT-Me (T_p) can be described by:

$$\mathrm{d}T_\text{a}/\mathrm{d}t = -k_\text{S}T_\text{a} - k_{\text{aIC}}T_\text{a} \quad \text{(S1.1)}$$

$$\mathrm{d}T_\text{p}/\mathrm{d}t = k_\text{S}T_\text{a} - k_{\text{pIC}}T_\text{p} \quad \text{(S1.2)}$$

where k_S, k_{aIC} and k_{pIC} denote the sensitization rate, the IC rate of the triplet state of anthracene, and the IC rate of the triplet state of IIDDT-Me, respectively. Solving equations S1.1 and S1.2 gives the following analytical expressions:

$$T_a = \exp[-(k_S + k_{aIC})t] \quad \text{(S2.1)}$$

$$T_p = \kappa * \{\exp(-k_{pIC}\,t) - \exp[-(k_S + \mathrm{k}_{aIC})\,t]\} \quad \text{(S2.2)}$$

where $\kappa = k_S/(k_S + k_{aIC} - k_{pIC})$ (S2.3)

In most cases, the sensitization process is faster than the IC process of the polymeric triplet state ($\kappa > 0$). The polymer signal builds up by the rate of $k_S + k_{aIC}$, and decays by the rate of k_{pIC}. But in this system, the triplet sensitization process is slower than the IC process of polymer triplet state ($\kappa < 0$), so the polymer signal builds up by the rate of k_{pIC}, and decays by the rate of $k_S + k_{aIC}$. The single-triplet lifetimeis identified by the rise of the polymer triplet signal (0.23μs).

In Supplementary Fig. 3, we represent the free triplet decay (T_p) for the raw data (black open circles) and fits (black solid lines) by plotting the multiplying the normalized kinetics by -1 then adding 1 to shift the signal in the y axis. The lifetime of triplet pairs generated by SF is shown with kinetic traces (red open circles) and fits(red solid lines). The lifetime of native single-triplet state (0.23 μs) of IIDDT-Me is ~ 100x longer than the lifetime of triplet state generated by SF.

Low Temperature Transient Absorption of IIDDT-Me in $CHCl_3$ Solution.

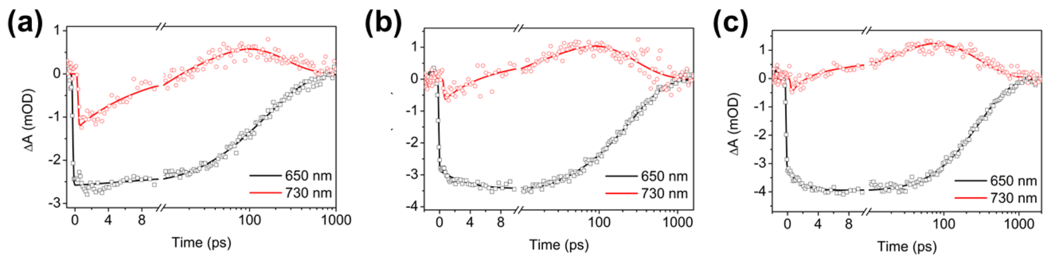

Supplementary Figure 4. The singlet fission dynamics are temperature dependent. Temperature dependent kinetics of IIDDT-Me in the $CHCl_3$ solution： (a) room temperature; (b) 270 K; (c) 255 K.

Supplementary Note 2. UV-Vis of IIDDT-Me in DCB at Elevated Temperature.

We confirm that our polymers are not aggregated in good solvents (chloroform and dichlorobenzene) since the attenuation spectra at elevated temperatures are unchanged.

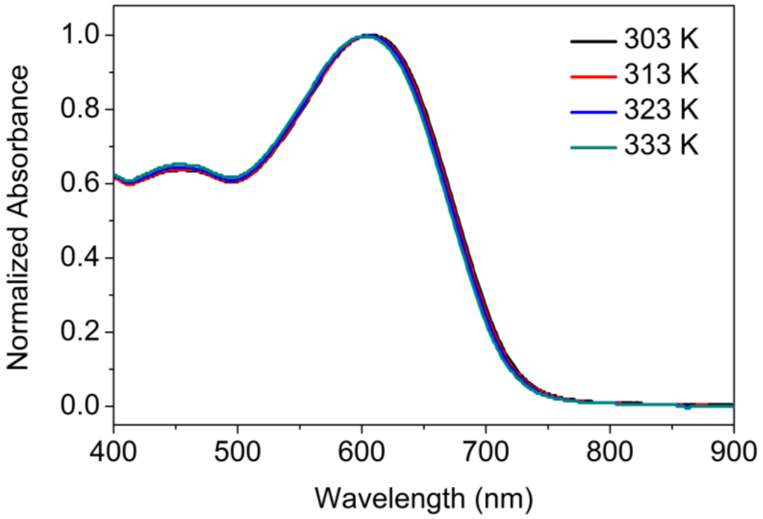

Supplementary Figure 5. UV-Vis absorption of IIDDT-Me in dichlorobenzene at elevated temperature.

Transient Absorption Data of IIDDT-Me under Aggregation

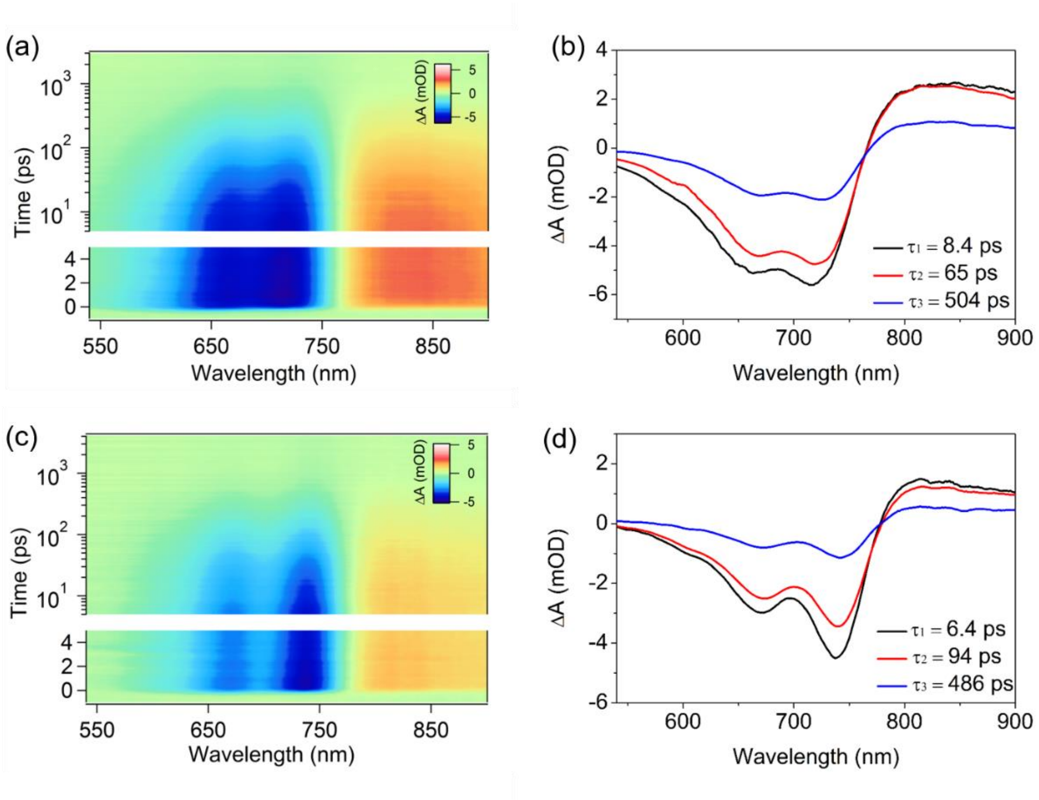

Supplementary Figure 6. **Films of IIDDT-Me do not exhibit measurable singlet fission.** (a) Transient absorption of IIDDT-Me in the $CHCl_3$ film is shown in a pseudo-color plot. (b) A sequential global analysis model of IIDDT-Me in the $CHCl_3$ film shows that no singlet fission occurs. (c) Transient absorption of IIDDT-Me in the TCE film is shown in a pseudo-color plot. (d) A sequential global analysis model of IIDDT-Me in the TCE film shows that no singlet fission occurs.

Supplementary Note 3. Fluence Dependent Transient Absorption Data of IIDDT-Me.

Our data are acquired in the low fluence regime, where the dynamics are independent of the pump intensity.

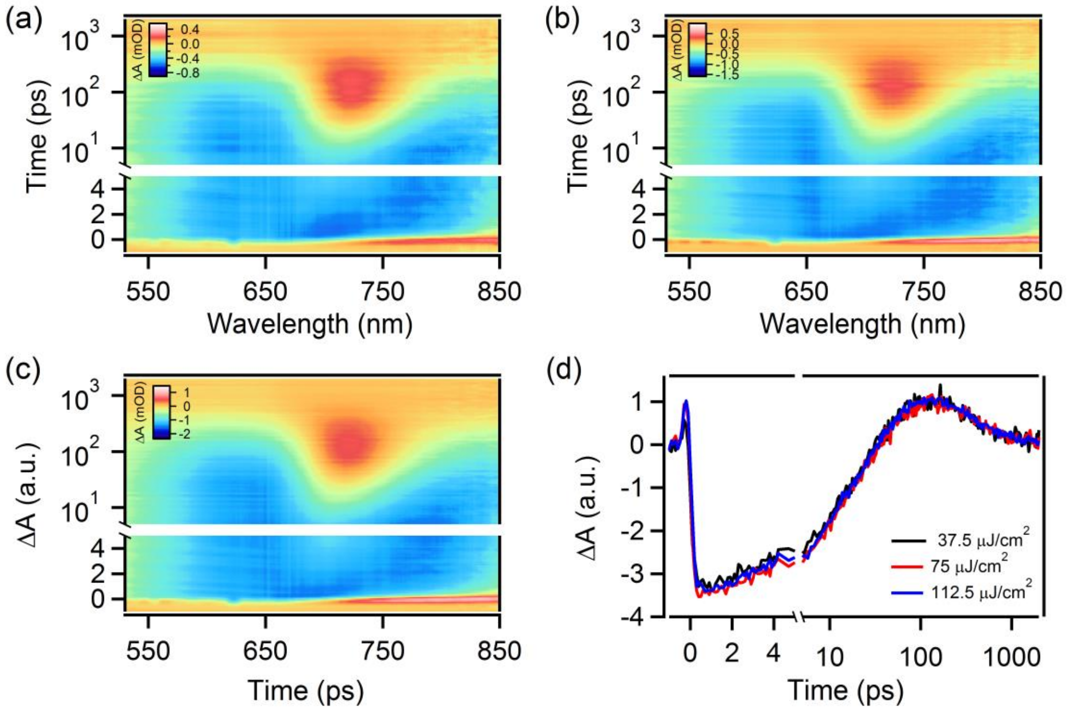

Supplementary Figure 7. Transient absorption of IIDDT-Me in the DCB solution at (a) 37.5 μJ cm^{-2}, (b) 75 μJ cm^{-2}, (c) 112.5 μJ cm^{-2} pump intensity are shown in pseudo-color plots, and the normalized kinetics at 720 nm is shown in (d).

Supplementary Note 4. Species Determination for Global Analysis

We have determined that there are three linearly independent components that must be included in our global analysis of the transient absorption and emission data for isolated IIDDT-Me chains. This number is determined using established protocols.[1,2]

A quick estimation of the number of species considers the number of non-zero singular values form singular value decomposition of the full data set. A graphical representation of the singular values (S matrix) is provided below in Supplementary

Fig. 8. It is clearly seen by inspection that the first two singular values are above the noise floor and that there are two additional ones that are difficult to distinguish from the noise floor. We conclude, in agreement with other authors,[3] that looking at the S matrix alone is not sufficient to determine the number of components.

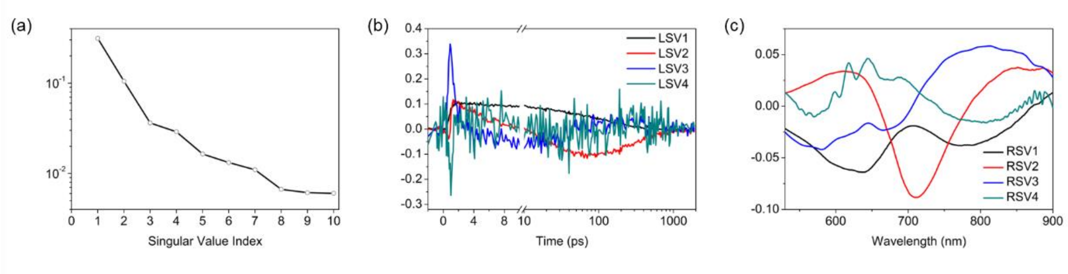

Supplementary Figure 8. (a) Singular values from singular value decomposition of transient absorption data. (b) The left singular vectors of transient absorption data. (c) The right singular vectors of transient absorption data.

Following this, the standard protocol is to reconstruct the data using an increasing number of components to determine when the reconstructed data no longer differs from the original data set by an amount larger than the noise. The two component analysis (Supplementary Fig. 9c and 9f) clearly exhibits regions where $\Delta\Delta A>0.5$, which is greater than the noise of the measurement by a considerable margin. In contrast, the three-component fit (Supplementary Fig. 9b and 9e) satisfactorily reproduces the data, suggesting we have chosen the appropriate rank=3 for our analysis.

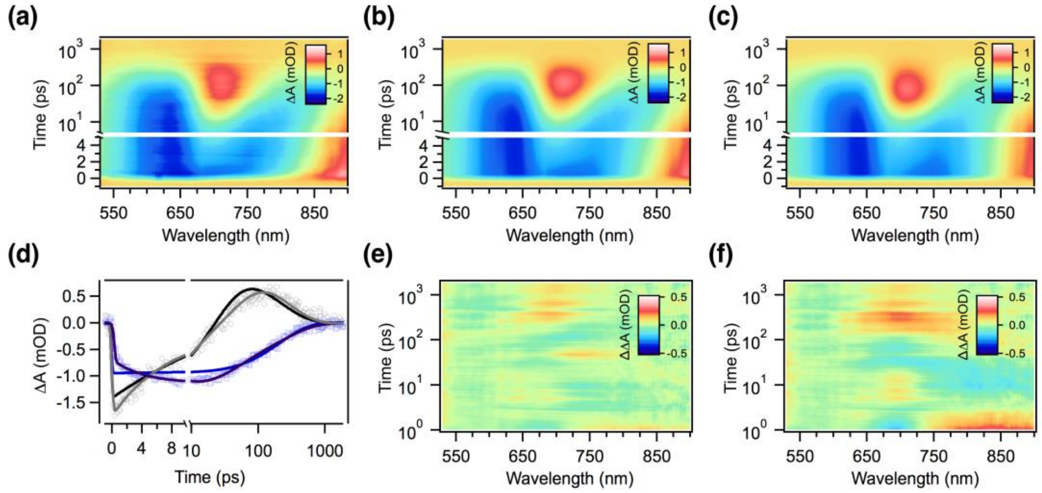

Supplementary Figure 9. (a) Transient absorption of IIDDT-Me in the diluted DCB solution is shown in a pseudo-color plot. (b) The global fitting of three exponentials. (c) The global fitting of two exponentials. (d) The dynamics taken from 700 nm (gray circles) and 810 nm (purple square), and the kinetic traces are shown in two time-constant fits (black line at 700 nm and blue line at 810nm) and three time-constant fits (gray line at 700 nm and purple line at 810 nm), respectively. (e) The residual transient absorption after global fitting of three exponentials. (f) The residual transient absorption after global fitting of two exponentials.

An alternative scheme has been suggested that utilizes a visual inspection of the principal kinetics (*U*) and principal spectra (*V*) scaled by the square root of the singular values $S^{1/2}$.[2] This treatment is show in Supplementary Fig. 10. Again, we conclude that rank 3 is the appropriate assignment, since the fourth scaled principal components are not significantly different than the zero lines.

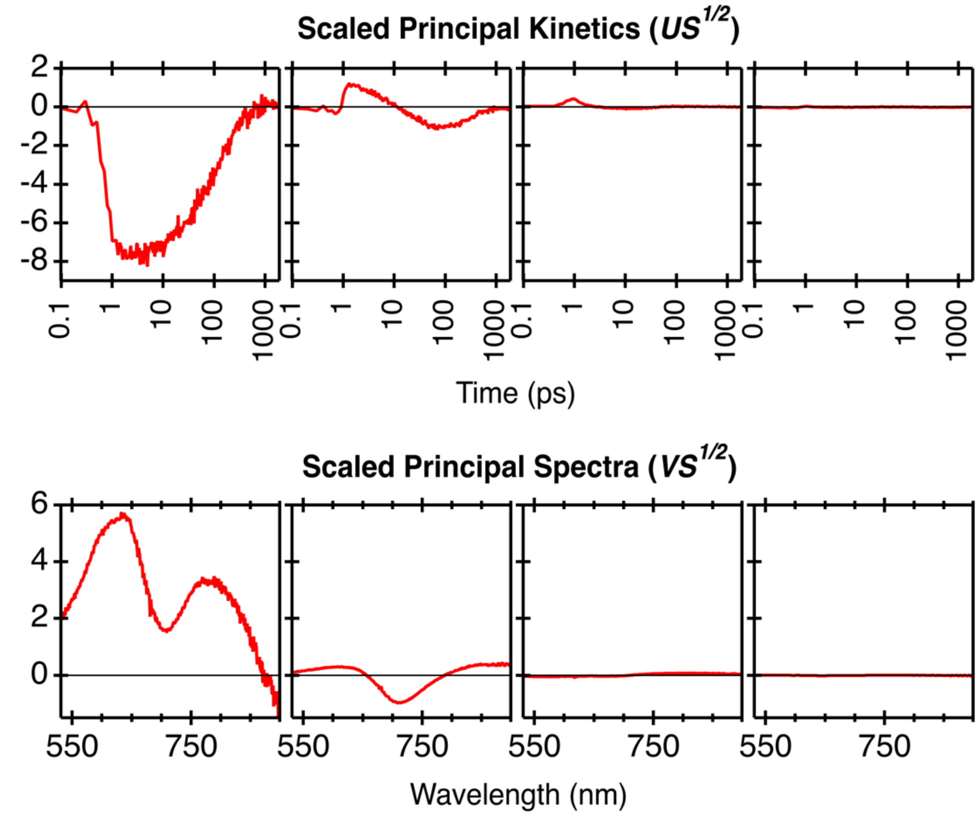

Supplementary Figure 10. Principal vectors scaled by the square root of the singular value. The 4th principal vectors are not distinguishable from the zero line.

A similar analysis was done for the transient emission data which similarly indicates three linear independent species are appropriate for a global analysis model. The leading terms in the S-matrix and corresponding left and right singular vectors for the TRPL data are shown in Supplementary Fig. 11.

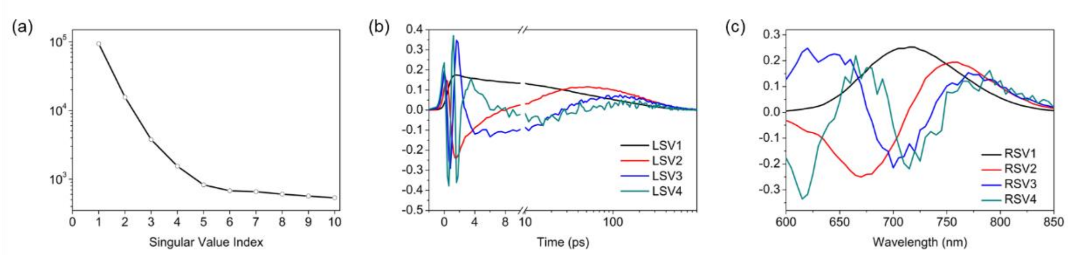

Supplementary Figure 11. (a) The singular value decomposition of transient fluorescence data. (b) The left singular vectors of transient fluorescence data. (c) The right singular vectors of transient fluorescence data.

Again, the S-matrix is not sufficient to determine the rank of the data set. Instead, we reconstruct the data using an increasing number of components to determine the point when the reconstructed data no longer differs from the original data set by an amount larger than the noise. Again, we find that a rank 3 sequential treatment is appropriate to describe our system.

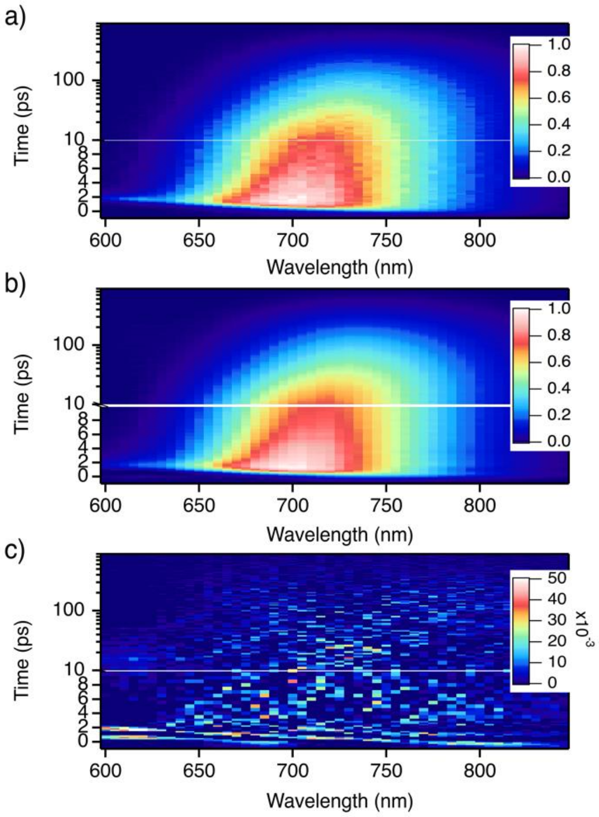

Supplementary Figure 12. (a) The raw TRPL data. (b) Reconstructed TRPL data generated from a 3 exponential global fit. (c) The residual TRPL signal from a 3 exponential sequential global analysis fit.

Supplementary References:

1. Henry, E. R. & Hofrichter, J. [8] Singular value decomposition: Application to analysis of experimental data. in *Methods in Enzymology* **210,** 129–192 (Academic Press, 1992).

2. DeSa, R. J. & Matheson, I. B. C. A Practical Approach to Interpretation of Singular Value Decomposition Results. in *Methods in Enzymology* **384,** 1–8 (Academic Press, 2004).

3. Satzger, H. & Zinth, W. Visualization of transient absorption dynamics – towards a qualitative view of complex reaction kinetics. *Chem. Phys.* **295,** 287–295 (2003).
